# Witnessing inter-parental violence in childhood and help-seeking behaviours in violence against women in Peru

**DOI:** 10.1186/s12889-024-18467-0

**Published:** 2024-04-12

**Authors:** Juan Carlos Bazo-Alvarez, Anthony Copez-Lonzoy, Miguel Ipanaqué-Zapata, Janina Bazalar-Palacios, Elizabeth López Rivera, Elaine C. Flores-Ramos

**Affiliations:** 1 Escuela de Medicina, Universidad Cesar Vallejo, Trujillo, Peru; 2 Research Department of Primary Care and Population Health, University College London, London, UK; 3 Unidad de Investigación en Bibliometría, Universidad San Ignacio de Loyola, Lima, Perú; 4 Vicerrectorado de Investigación, Universidad Privada Norbert Wiener, Lima, Peru; 5 Universidad Científica del Sur, Miraflores, Peru; 6 PSYCOPERU Peruvian Institute for Psychological and Psychosocial Research, Lima, Peru; 7 Centre on Climate Change & Planetary Health, London School of Hygiene and Tropical Medicine, London, UK; 8 Stanford Center for Innovation in Global Health, Stanford Woods Institute for the Environment, Stanford, USA

**Keywords:** Peru, Witnessing violence, Help-seeking behaviours, Violence against women

## Abstract

**Background:**

Violence against women (VAW) severely impacts their physical and mental health. In some cultures, women can normalize certain types of violence if they were linked to home models in childhood and, eventually, do not seek for help in adulthood. We aimed to determine, in Peruvian women, (1) the association between witnessing violence in their family of origin and VAW experienced in adulthood, (2) the extent to which women who have experienced VAW seek some help, and (3) identify VAW prevalence by Peruvian region.

**Methods:**

Cross-sectional study of secondary data obtained from the 2019 National Demographic and Family Health Survey (ENDES). The outcome was VAW (psychological, physical and sexual violence), whereas the exposure was witnessing violence in the home of origin. Help-seeking behavior was a secondary outcome, for which VAW was the exposure. Prevalence ratios (PR) were estimated to assess both associations, unadjusted and adjusted for covariates (aPR).

**Results:**

Data from 14,256 women aged 15 to 49 years were analysed. 51.5% reported having experienced VAW and 43.8% witnessed violence in the home of origin during childhood. Witnessing inter-parental violence in childhood was associated with psychological violence aPR = 1.25 (95% CI: 1.17–1.33), physical aPR = 1.52 (95% CI: 1.38–1.67), and sexual aPR = 1.99 (95% CI: 1.57–2.52). Women who have experienced both types of violence (physical and sexual) were more likely to help-seeking (aPR = 1.30, 95% CI: 1.14–1.50) than women suffering only one type of violence.

**Conclusion:**

Women who reported having witnessed home violence in their childhood are more likely to experience Violence Against Women (VAW) by their current partner. Physical and sexual violence with a current partner was more associated with witnessing inter-parental violence in childhood, and when physical and sexual violence jointly occurred women were more help-seeking. The southern region of Peru is identified as an area of high vulnerability for women. It is crucial to promote educative and community-based programs aimed at the prevention and early recognition of VAW.

**Supplementary Information:**

The online version contains supplementary material available at 10.1186/s12889-024-18467-0.

## Background

Violence Against Women (VAW) comprising Intimate parent violence (IPV) and Sexual Violence is a complex societal problem and the most common form of violence partner [1]. The evidence base on the extent of the problem, the risk factors and the profound mental and physical consequences that violence brings to women’s lives and wellbeing are well documented [1–5]. Additionally, VAW is costly for communities and societies, preventing women from participating fully in labour activities [1]. The World Health Organization (WHO, 2021) reports that 1 in 3 women have experienced at least some type of violence (psychological, physical and/or sexual) at some point in their lives [1, 6]. Approximately 27% of women between the ages of 15 and 49 have been in a relationship where they have been victims of violence [1]. Regardless of its broad distribution globally, there is still a considerable variation at the regional and national levels, without much understanding of why this occurs. For example, by 2017, in Latin America, 30% of women have experienced some type of violence. The countries leading the highest rates of psychological and sexual violence were Bolivia (58.5%), Ecuador (40.4%), Colombia (33.1%) and Peru (31.2%) [7].

Population-level exposure to other forms of violence (e.g. witnessing parental violence or child abuse) and patriarchal social norms (e.g. through harmful use of alcohol, food insecurity, stigma, maintenance of roles of gender, reinforcement of authority and position by the partner) result in high levels of VAW who are responsible for potential risk factors [8]. Witnessing domestic violence in the family home of origin is an essential risk marker for the development of VAW [9]. This risk marker increases the likelihood of violence and victimization with the present partner by 44% and the likelihood of repeating this behavior in adulthood by 2–6 times [10–12].

Despite the evidence base for this association between witnessing parental violence and VAW, the existence of a large number of studies focused on factors at the individual level (e.g. relational, economic, behavioural, experiential factors, etc.) in our knowledge, the structural and contextual mechanisms (e.g. community-level risk factors and widespread poverty in the indigenous population and inequities) still need to be further identified, especially in environments with a high-prevalence of VAW such as Peru [7, 8, 13, 14]. Also, multiple overlapping factors are responsible for this higher frequency of VAW in these settings [8].

These episodes of violence can impact the intention and behaviour of help-seeking. These behaviours make it possible to recognise and be aware of a problem that may require the intervention of another person (or institution) [15, 16]. In addition, it helps maintain emotional competencies that generate previous positive experiences and greater ease for professional care by increasing mental health literacy skills. Likewise, there is a greater tendency to seek informal help (for example, Family and friends) [16–18]. However, the deficit of this help-seeking behaviour in VAW is a multifactorial loop due to the severity of the violence added to individual factors (e.g. age of abuse, economic stress, education, presence of children) normalising this problem [15–17].

Also, the lack of intention to help-seeking in VAW episodes is compounded by external and internal barriers [19]. At the superficial level, these barriers are shown as structural components of the failed experience in violence care centers (e.g. few crisis centers, insufficient sanctions and low probability of prosecuting the aggressors) [20] and internal factors such as the low perception of violence in vulnerable conditions (e.g. financial dependence, gestation periods, number of children and concerns about immigration laws). These factors usually determine adherence to violence prevention programs and only maintain 27% [11]. However, there are no conclusive results on the different conditions of exposure to violence (one or more types of violence) that may facilitate the search for help-seeking.

To our knowledge, this is the first study focusing on witnessing in origin-place violence and help-seeking using nationally-representative survey data from a highly prevalent country of VAW such as Peru. It builds on previous studies that have assessed intimate partner violence using a secondary data approach [21–23]. Our study aims were to determine, in Peruvian women, (1) the association between witnessing inter-parental violence in childhood and VAW experienced in adulthood and (2) the extent to which women who have experienced VAW help-seeking, and (3) identify VAW prevalence by Peruvian region.

## Methods

### Study design

This is a secondary analysis of the 2019 dataset of the Demographic and Family Health Survey (DHS), ENDES in Peru [24]. The 2019 data were used because they were not exposed to possible selection bias (e.g., COVID-19 pandemic). ENDES is an annual survey with cluster sampling, stratified and nationally representative conducted by the National Institute of Statistics and Informatics (INEI). Likewise, the ENDES is composed of questionnaires at the household, individual (information on women of childbearing age) and health levels to present knowledge on health indicators in the general Peruvian population [25]. Finally, the ENDES uses a two-stage probabilistic sampling, stratified according to rural and urban areas in the 25 regions of Peru, generating results with national representativeness [25]. For this study, we used information from the questionnaire applied to women of childbearing age and households.

### Setting and population

**Fig. 1:**
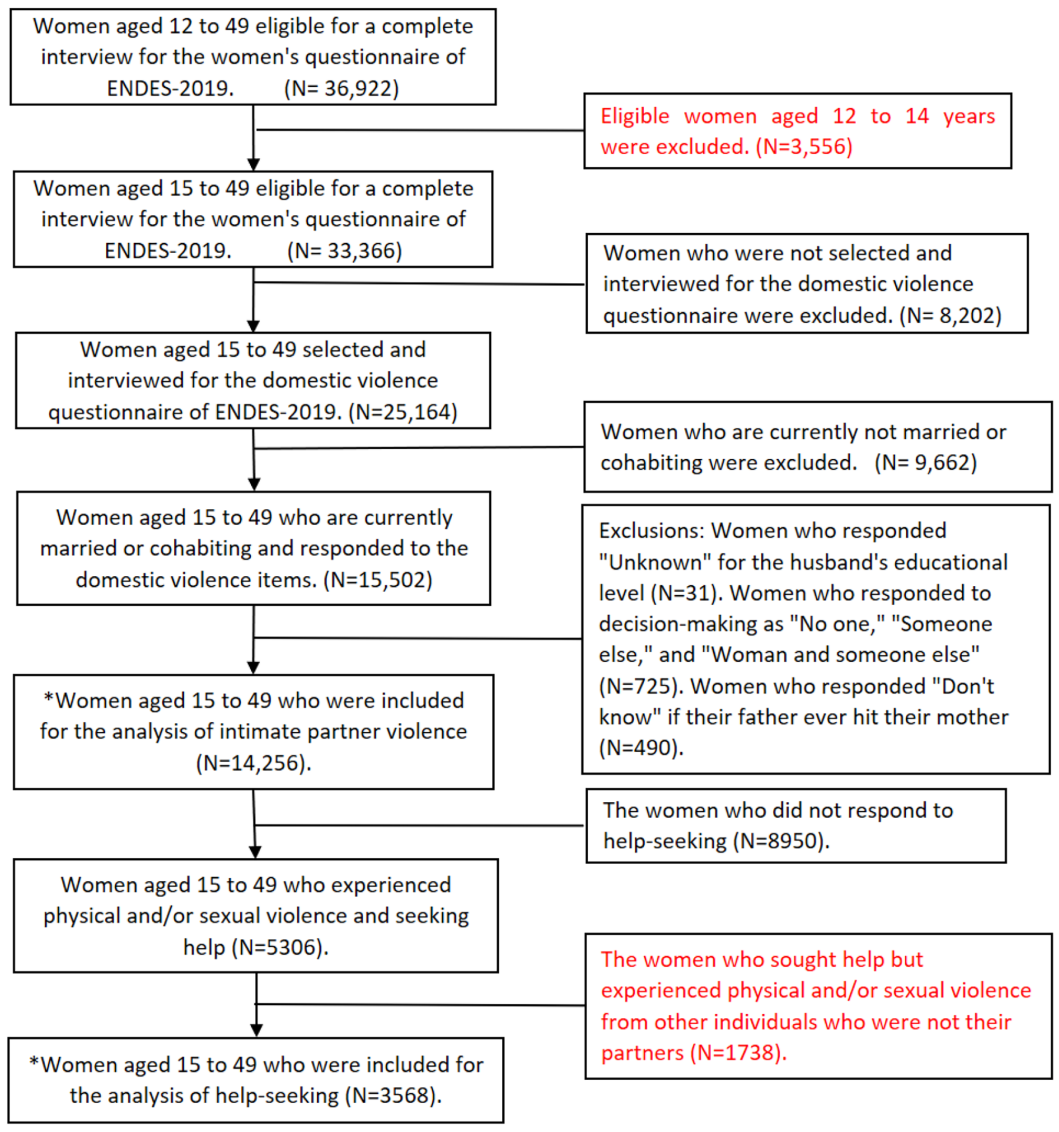
Flowchart of inclusion of participants in the analysis

The DHS reported that the number of women of childbearing age (15 to 49 years) who responded to the full questionnaire was 36,922 in 2019 [25]. From this, we obtained an initial sample of women who responded to VAW, where we excluded women who did not have privacy for this section of violence and missing data in the variables of our study, obtaining a sample of 14,256 women of childbearing age. Finally, for the analysis of seeking help for violence, the questions on physical and sexual violence were taken into account, so only women who reported suffering some type of physical and/or sexual violence were selected, which reduced the sample to 3,568 women of childbearing age (see Fig. 1). This study did not show missing data.

### Study variables

In the study, the main variables of the study were VAW and help-seeking as dependent variables and witnessing violence, and physical and/or sexual violence as independent variables. VAW was presented through indicators of psychological, physical and sexual violence exerted by the current partner, this instrument was constructed from 18 questions with a dichotomous scale (Yes/No) on psychological [9], physical [7] and sexual [2] aggression. Help-seeking for episodes of violence perpetrated by the partner was obtained from the self-report of a complaint or request for support from the woman to another person after the aggression occurred, taking into account the questions directed towards physical and sexual violence. Witnessing inter-parental violence in childhood was obtained from the woman knowledge of the violence perpetrated by her father against her mother [26, 27]. This item is one of the important indicators of the construct of witnessing interparental violence (witnessing violence from the father towards the mother and vice versa) [28]. The children who observed episodes of inter-parental violence occurred in the home of the victims [26, 27, 29]. This question has already been used as part of the witnessing violence survey [28] This component helps explain the etiology of the cycle of violence related to the couple [28, 30]. Finally, the variable physical and/or sexual violence obtained from the indicators of physical and sexual VAW to know the number of forms of violence perceived by the partner. It was presented in two response categories (physical or sexual violence and both types of violence). The other main variables also presented two response categories (Yes and Not).

The study covariates were obtained through the directed acyclic graph (DAG), such as the difference in educational level understood as the imbalance in the access and completion of education by one of the members of the couple generating three response categories (both with the same level, the woman with higher level and the man with higher level), decision making (joint decision, woman’s predominant decision, man’s predominant decision and split decision), couple communication (Yes/No) and respect by the partner (Yes/Not). Other variables included in the study were age (categorised as 15–28, 29–35 and 36–49 years), educational level (recategorised as no/primary level, secondary level and higher level), marital status (categorised as married and cohabiting), economic level (recategorised as very poor/poor, medium and very rich/rich), currently working (No/Yes), area of residence (recategorised as Coast, Highlands and Jungle), place of origin (urban and rural), has health insurance (No/Yes), partner drinks alcohol (recategorised into Does not drink, drinks but does not get drunk, Drinks and gets drunk sometimes, and Drinks and gets drunk often), age at the start of cohabitation (recategorised into ≤ 18, 19–21 and 22 or older), number of children (recategorised into None, 1–2 children, 3–4 children, 5 or more children) and partner respect (Yes/No). All information on the study variables from the ENDES and indicators of validity and reliability of the variables generated are detailed in the supplementary material 1. In the study, only women who experienced violence from their current partner were included.

### Statistical analysis

For the study variables, descriptive analyses were performed, reporting frequencies and percentages. In addition, prevalence maps were implemented according to types of violence (psychological, physical, sexual and global) for all the regions of Peru.

For the first objective, we modelled the occurrence of current violence (binary) by fitting Poisson regression models with robust variance [31, 32]. In these models, the primary exposure witnessing violence. We fitted four different models, one per type of current violence (psychological, physical and sexual). The Poisson models allowed us to estimate prevalence ratios (PR), unadjusted and adjusted for the covariates associated with the outcome in the unadjusted models.

The same regression models were used for the second objective. We evaluated the occurrence of help-seeking (binary) in an exploratory model. This model was divided into two phases, the first used a list of covariates so that they are included in the nested model, and the significance level was relaxed to 0.20. In the second phase, we included only the sequentially significant variables in the Log-likelihood test (*p* <.05). In addition, to identify the concentration of types of violence by region, we use geolocation maps.

All analyses were adjusted using the weighting factor according to the DHS complex sampling [10], using the “svy” command in Stata 16.1 (Stata Corporation, College Station, Texas, USA), while we generated maps using ArcGIS 10.8 (ESRI Inc., Redlands, CA, USA) and to obtain the psychometric properties of the instrument of violence we use Mplus7 (see supplemental material 1).

## Results

**Fig. 2:**
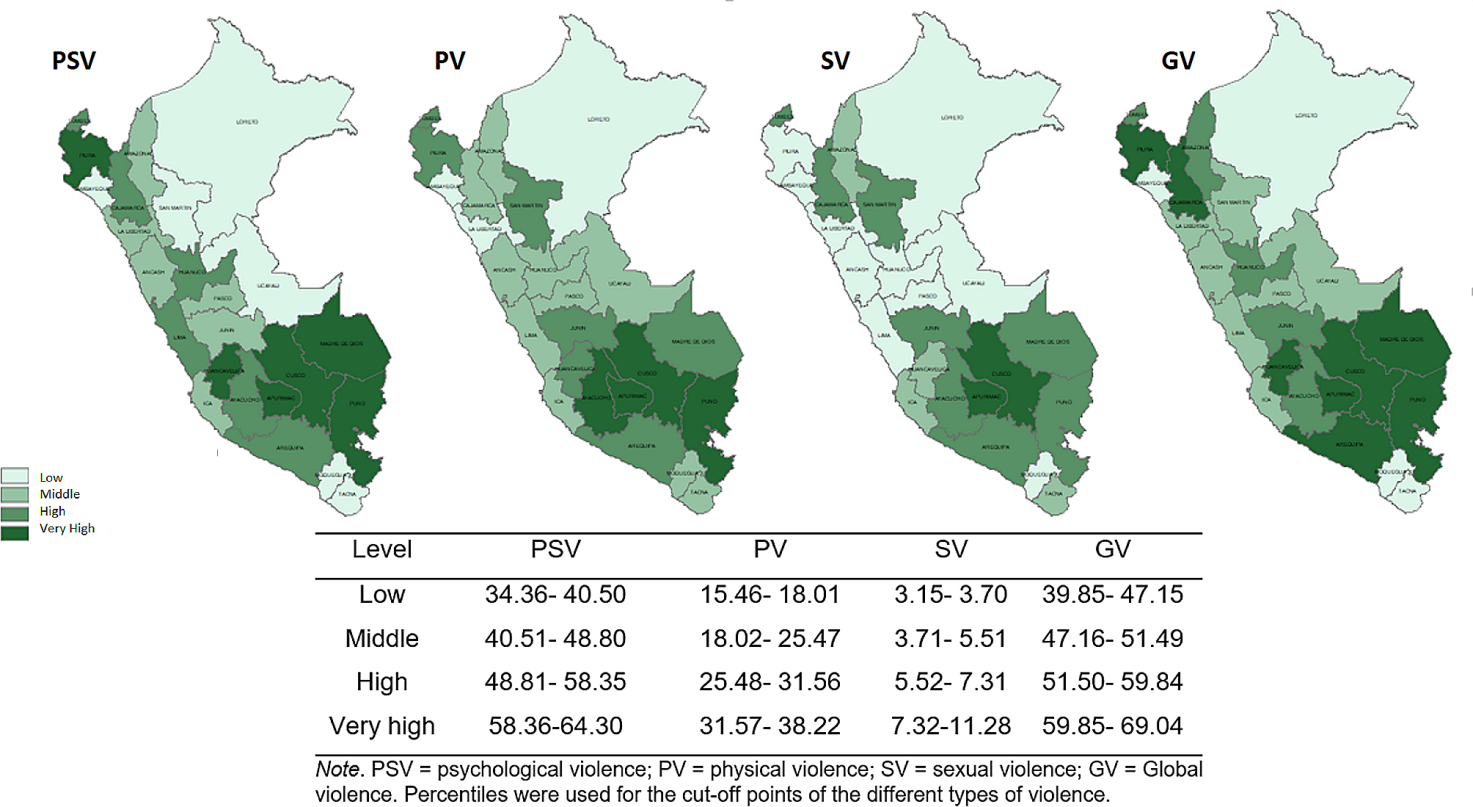
Maps according to types of violence against women by region

The characteristics of the sample reported that the mean age was 34.7 (95% CI 34.4–34.9). 6915 48.5%) stated that they were victims of VAW where 6733 (47.2%) suffered psychological violence, 35,016 (24.6%) physical violence and 619 (4.3%) sexual violence. 35% had a high school education, 65.1% were cohabitants, and 56.4% had only 1–2 children (Table 1). The provinces with the highest prevalence of psychological/verbal, physical, sexual and global violence were: Puno, Apurimac and Cusco, all located in the southern highlands of Peru (Fig. 2).

**Table 1:** Descriptive characteristics of women of reproductive age, ENDES 2019 (*N* = 14,256)

	N (%)	[IC95%]
**Age**		
15–28	3567(25%)	23.92-26.16%
29–35	4700(33%)	31.54-34.43%
36–49	5989(42%)	40.48-43.56%
**Education level**		
No Education/Primary	3087(21.7%)	20.54-22.81%
Secondary	6178(43.3%)	41.84-44.84%
Higher education	4991(35%)	33.47-36.58%
**Marital Status**		
Married	4981(34.9%)	33.39-36.52%
Cohabiting	9275(65.1%)	63.48-66.61%
**Economic level**		
Very Poor/Poor	6252(43.9%)	42.35-45.37%
Medium	3032(21.3%)	20.01-22.58%
Rich/Very rich	4972(34.9%)	33.28-36.5%
**Currently working**		
Not	5268(37%)	35.55-38.38%
Yes	8988(63%)	61.62-64.45%
**Area of residence**		
Coast	8412(59%)	57.57-60.43%
Highlands	3648(25.6%)	24.3-26.93%
Jungle	2195(15.4%)	14.44-16.41%
**Place of origin**		
Urban	10,980(77%)	76.09-77.92%
Rural	3276(23%)	22.08-23.91%
**Health Insurance**		
Not	3064(21.5%)	20.2-22.85%
Yes	11,192(78.5%)	77.15-79.8%
**Differences in educational level**		
Both with the same level	9242(64.8%)	63.3-66.32%
Women with a higher level	1988(13.9%)	12.91-15.05%
Men with a higher level	3026(21.2%)	20.06-22.44%
**Number of children**		
None	1113(7.81%)	6.78-8.97%
1–2 Children	8047(56.4%)	54.87-58.01%
3–4 or more children	3996(28%)	26.74-29.35%
5 or more	1100(7.72%)	7.09-8.39%
**Age of start of cohabitation**		
≤ 18 years old	4998(35.1%)	33.72-36.42%
19–21 years old	4388(30.8%)	29.47-32.11%
22 and over	4870(34.2%)	32.64-35.72%
**Partner drinks alcohol**		
Does not drink	3227(22.6%)	21.23-24.11%
Drinks but does not get drunk	2891(20.3%)	18.93-21.7%
Drinks and gets drunk sometimes	7701(54%)	52.36-55.67%
Drinks and gets drunk often	437(3.1%)	2.61-3.6%
**Decision making**		
Joint decision	4948(34.7%)	33.22-36.23%
Woman’s predominant decision	7448(52.2%)	50.67-53.81%
Man’s predominant decision	847(5.94%)	5.32-6.62%
Split decision	1013(7.11%)	6.37-7.93%
**Couple communication**		
Not	678(4.76%)	4.15-5.46%
Yes	13,578(95.2%)	94.54-95.85%
**Respect by the partner**		
Not	586(4.11%)	3.53-4.78%
Yes	13,670(95.9%)	95.22-96.47%
**Psychological Violence**		
Not	7523(52.8%)	51.24-54.3%
Yes	6733(47.2%)	45.7-48.76%
**Physical Violence**		
Not	10,750(75.4%)	74.13-76.64%
Yes	3506(24.6%)	23.36-25.87%
**Sexual Violence**		
Not	13,637(95.7%)	95.05-96.19%
Yes	619(4.34%)	3.81-4.95%
**Global Violence**		
Not	6915(48.5%)	47.03-49.99%
Yes	7341(51.5%)	50.01-52.97%
**Parental aggression**		
Not	13,375(93.9%)	93.02-94.65%
Yes	871(6.11%)	5.35-6.98%
**Witnessing of violence**		
Not	8218(57.6%)	56.1-59.18%
Yes	6038(42.4%)	40.82-43.9%
Help Seeking*		
No	2007(56.32%)	53.3-59.16%
Yes	1561(43.8%)	40.84-46.72%

Note: *n* = 14,256. * women who responded experienced physical and/or sexual violence by their partner (*n* = 3,568)

The row model presents the result for the three types of violence (psychological, physical and sexual) perpetrated by the current partner. The prevalence in women who were witnessing inter-parental violence in childhood and experienced some type of violence (psychological, physical or sexual) was between 1.25 and 1.99 more than the group of women who witnessing inter-parental violence in childhood. In addition, the variables couple communication, decision making, alcohol consumption by the partner, age at the beginning of cohabitation, number of children, area of residence and educational level were statistically significant (*p* <.05). Therefore, they were included in the adjusted model (Table 2).

**Table 2:** Association between witnessing inter-parental violence in childhood and VAW, ENDES 2019 (*N* = 14,256)

	Psychological				Physical				Sexual			
	PR (IC 95%)	*p*	aPR (IC 95%)	*p*	PR (IC 95%)	*p*	aPR (IC 95%)	*p*	PR (IC 95%)	*p*	aPR (IC 95%)	*p*
**Witnessing inter-parental violence**												
Not	ref.		ref.		ref.		ref.		ref.		ref.	
Yes	1.33(1.24–1.42)	< 0.001	1.25(1.17–1.33)	< 0.001	1.68(1.52–1.86)	< 0.001	1.52(1.38–1.67)	< 0.001	2.36(1.82–3.05)	< 0.001	1.99(1.57–2.52)	< 0.001
**Age**												
15–28	ref.				ref.		ref.		ref.		ref.	
29–35	0.96(0.89–1.04)	0.337			1.12(0.98–1.27)	0.091	1.06(0.93–1.21)	0.396	1.56(1.12–2.17)	0.009	1.43(0.99–2.07)	0.056
36–49	1.04(0.97–1.12)	0.263			1.35(1.19–1.53)	< 0.001	1.21(1.05–1.39)	0.009	2.15(1.6–2.88)	< 0.001	1.65(1.15–2.36)	0.006
**Level Education**												
No Education/Primary ref.	ref.		ref.		ref.		ref.		ref.		ref.	
Secondary	1.08(1–1.16)	0.04	1.10(1.01–1.19)	0.022	0.95(0.84–1.06)	0.354	1.05(0.91–1.21)	0.494	0.72(0.56–0.94)	0.016	1.10(0.85–1.42)	0.474
Higher education	0.82(0.75–0.90)	< 0.001	0.95(0.86–1.06)	0.344	0.65(0.56–0.75)	< 0.001	0.91(0.74–1.12)	0.397	0.42(0.3–0.61)	< 0.001	1.25(0.82–1.92)	0.301
**Marital Status**												
Married	ref.		ref.		ref.				ref.			
Cohabiting	1.12(1.04–1.2)	0.003	1.07(1–1.14)	0.061	1.10(0.98–1.24)	0.12			1.16(0.88–1.52)	0.29		
**Economic level**												
Very Poor/Poor	ref.		ref.		ref.		ref.		ref.		ref.	
Medium	1.03(0.95–1.11)	0.506	1.05(0.97–1.14)	0.207	0.95(0.85–1.08)	0.439	1.02(0.88–1.19)	0.755	0.81(0.57–1.16)	0.256	1.54(0.94–2.54)	0.087
Rich/Very rich	0.89(0.82–0.96)	0.003	1.02(0.93–1.11)	0.674	0.79(0.69–0.90)	< 0.001	1.05(0.89–1.23)	0.561	0.49(0.32–0.74)	0.001	1.52(0.91–2.52)	0.108
**Currently working**												
Not	ref.		ref.		ref.		ref.		ref.		ref.	
Yes	1.08(1.02–1.15)	0.015	1.08(1.02–1.15)	0.013	1.25(1.12–1.38)	< 0.001	1.18(1.06–1.3)	0.002	1.57(1.16–2.11)	0.003	1.31(1.02–1.68)	0.032
**Area of residence**												
Coast	ref.		ref.		ref.		ref.		ref.		ref.	
Highlands	1.15(1.07–1.22)	< 0.001	1.07(1–1.15)	0.04	1.38(1.24–1.54)	< 0.001	1.28(1.14–1.43)	< 0.001	1.7(1.27–2.27)	< 0.001	1.13(0.84–1.52)	0.411
Jungle	0.92(0.85–0.99)	0.032	0.84(0.78–0.91)	< 0.001	1.24(1.11–1.4)	< 0.001	1.13(1.01–1.27)	0.038	1.5(1.1–2.04)	0.01	0.97(0.73–1.28)	0.816
**Place of origin**												
Urban	ref.				ref.		ref.		ref.		ref.	
Rural	0.97(0.91–1.03)	0.335			1.11(1.02–1.21)	0.021	0.9(0.82–1)	0.046	1.61(1.28–2.01)	< 0.001	1.15(0.86–1.53)	0.339
**Health Insurance**												
Not	ref.				ref.				ref.			
Yes	1.05(0.96–1.15)	0.247			1.1(0.96–1.26)	0.181			1.26(0.87–1.83)	0.223		
**Differences in educational level**												
Both with the same level	ref.				ref.				ref.			
Woman with a higher level	1(0.91–1.10)	0.987			1.08(0.93–1.26)	0.323			1.23(0.84–1.79)	0.281		
Men with a higher level	1.04(0.97–1.13)	0.258			1.10(0.97–1.24)	0.140			1.16(0.88–1.53)	0.287		
**Number of children**												
None	ref.		ref.		ref.		ref.		ref.		ref.	
1–2 Children	1.13(0.94–1.35)	0.185	1.04(0.89–1.23)	0.61	1.88(1.24–2.84)	0.003	1.57(1.06–2.32)	0.025	1.5(0.46–4.92)	0.505	1.09(0.38–3.17)	0.869
3–4 or more children	1.31(1.09–1.57)	0.004	1.12(0.95–1.32)	0.191	2.73(1.79–4.15)	< 0.001	1.85(1.23–2.8)	0.003	3.42(1.04–11.31)	0.044	1.54(0.5–4.7)	0.45
5 or more	1.33(1.1–1.6)	0.003	1.10(0.92–1.32)	0.277	3.04(1.99–4.63)	< 0.001	1.83(1.19–2.79)	0.005	5.39(1.63–17.84)	0.006	1.70(0.54–5.35)	0.366
**Age of start of cohabitation**												
19–21 años	ref.		ref.		ref.		ref.		ref.		ref.	
≤ 18 years old	1.06(0.99–1.13)	0.093	1.04(0.97–1.11)	0.247	1.15(1.03–1.28)	0.012	1.09(0.98–1.21)	0.098	1.51(1.16–1.98)	0.003	1.29(1.01–1.66)	0.04
22 and over	0.82(0.75–0.9)	< 0.001	0.88(0.81–0.96)	0.005	0.73(0.62–0.85)	< 0.001	0.80(0.69–0.94)	0.006	0.72(0.49–1.06)	0.098	0.78(0.54–1.13)	0.188
**Partner drinks alcohol**												
Does not drink	ref.		ref.		ref.		ref.		ref.		ref.	
Drinks but does not get drunk	0.90 (0.8–1.01)	0.081	0.93(0.83–1.04)	0.184	1.14(0.93–1.4)	0.22	1.24(1.02–1.52)	0.034	0.59(0.33–1.07)	0.082	0.74(0.41–1.33)	0.316
Drinks and gets drunk sometimes	1.20 (1.09–1.31)	< 0.001	1.16(1.06–1.26)	0.001	1.51(1.30–1.76)	< 0.001	1.42(1.23–1.65)	< 0.001	1.91(1.28–2.86)	0.002	1.74(1.17–2.59)	0.006
Drinks and gets drunk often	2.13(1.94–2.33)	< 0.001	1.69(1.53–1.87)	< 0.001	4.35(3.7–5.13)	< 0.001	2.9(2.44–3.44)	< 0.001	11.49(7.19–18.37)	< 0.001	4.57(2.69–7.79)	< 0.001
**Decision making**												
Joint decision	ref.		ref.		ref.		ref.		ref.		ref.	
Woman’s predominant decision	1.24(1.15–1.33)	< 0.001	1.15(1.07–1.23)	< 0.001	1.57(1.4–1.77)	< 0.001	1.37(1.23–1.53)	< 0.001	2.25(1.52–3.34)	< 0.001	1.53(1.06–2.23)	0.025
Man’s predominant decision	1.22(1.07–1.38)	0.002	1.18(1.05–1.32)	0.006	1.27(1.02–1.59)	0.03	1.14(0.91–1.43)	0.25	1.79(1.06–3.02)	0.03	1.28(0.79–2.07)	0.322
Split decision	1.34(1.21–1.48)	< 0.001	1.29(1.17–1.43)	< 0.001	1.48(1.23–1.78)	< 0.001	1.34(1.13–1.58)	0.001	2.06(1.24–3.42)	0.005	1.61(1.00–2.59)	0.049
**Couple communication**												
Yes	ref.		ref.		ref.		ref.		ref.		ref.	
Not	1.75(1.64–1.88)	< 0.001	1.19(1.07–1.33)	0.002	2.29(2–2.62)	< 0.001	1.04(0.88–1.23)	0.625	6.94(5.17–9.3)	< 0.001	1.13(0.78–1.65)	0.511
**Parental agression**												
Not	ref.		ref.		ref.		ref.		ref.			
Yes	1.35 (1.21–1.51)	< 0.001	1.29 (1.15–1.43)	< 0.001	1.48 (1.23–1.78)	< 0.001	1.37 (1.15–1.63)		1.60 (0.89–2.88)	0.11		
**Respect by the partner**												
Yes	ref.		ref.		ref.		ref.		ref.		ref.	
Not	1.93(1.81–2.05)	< 0.001	1.43(1.29–1.60)	< 0.001	3.01(2.67–3.40)	< 0.001	1.97(1.69–2.29)	< 0.001	12.04(9.41–15.41)	< 0.001	6.07(4.36–8.44)	< 0.001

Note: *n* = 14,256. *p* <.001, PR: crude prevalence ratio, aPR: adjusted prevalence ratio. The final model was adjusted by variables that were associated with the crude model for the three types of violence: age, educational level, marital status, socioeconomic level, current working, area of residence, place of origin, health insurance, the difference in educational level (imbalance education access), number of children, age of beginning of cohabitation, partner drinks alcohol, decision making in the couple, couple communication, parental aggression and respect by the partner

Compared to the crude model, the adjusted model showed a decrease in prevalence between 7% and 37% concerning the women who did not witness the types of violence. It is essential to mention that for women who witnessed violence by their father towards their mother, it was associated with 1.25 (95% CI 1.17 to 1.33) times more often experience psychological violence, even up to 1.99 (95% CI 1.57 to 2.52) times in experiencing sexual violence with their current partners (Table 2). However, this probability was slightly reduced in the case of global violence 1.23 (95% CI 1.16 to 1.30) and for the presentation of both types of violence (physical and sexual) it increased by 33% (see supplemental material 1). In addition, the variables of partner respect, decision making and alcohol consumption by the partner presented a more significant impact and increased prevalence of the all types of violence. They were significant for all types of violence (*p* <.05).

The association of physical and/or sexual violence with support-seeking was posited from an exploratory standpoint to be statistically significant (*p* <.05). The adjusted model indicated that women who experienced both types of violence had a 1.30 higher prevalence of support-seeking compared to women who experienced only one type of violence (95% CI 1.14 to 1.50) (Table 3).

**Table 3:** Association between Physical or Sexual violence and help-seeking with the adjusted and stepwise model, ENDES 2019 (*N* = 3,568)

	Help-seeking		Adjusted Model^1^	
	No n(%)	Si n(%)	aPR (IC 95%)	*p*
**Physical or Sexual Violence**				
Only physical or sexual violence	1760(87.7%)	1257(80.5%)	ref.	
Both types of violence	247(12.3%)	304(19.5%)	1.30 (1.14–1.50)	< 0.001
**Level Education**				
No Education/Primary	527(26.3%)	376(24%)	ref.	
Secondary	900(44.8%)	799(51.2%)	1.13 (0.99–1.29)	0.070
Higher	580(28.9%)	386 (24%)	1.00(0.83–1.21)	0.998
**Marital Status**				
Married	699(34.8%)	470(30.1%)	ref.	
Cohabiting	1308(65.2%)	1091(69.9%)	1.10 (0.95–1.27)	0.212
**Couple communication**				
Yes	1825(91%)	1375(88.1%)	ref.	
Not	182(9%)	186(11.9%)	1.07(0.88–1.30)	0.527

Note: *n* = 3568. aPR: adjusted prevalence ratio. *p* <.001. variables included in the stepwise model: physical or sexual violence, partner drinking alcohol and age of onset of cohabitation. ^1^The model was adjusted level education, marital status and couple communication (*p* <.001). Only variables that maintained *p* <.20 in the raw model were included

## Discussion

We assessed the association between witnessing violence in the home of origin and VAW. Witnessing inter-parental violence in childhood was associated with psychological, physical, and sexual violence with the current partner. In addition, suffering both types of violence (physical and sexual) increased the likelihood of help-seeking. The southern region of Peru condensed the highest prevalence of VAW (psychological, physical and sexual).

In Peru, the presence of psychological violence doubles the results evaluated in Latin America [5], generating a cumulative effect on health and possible economic and social effects on victims of violence [33, 34]. Likewise, witnessing inter-parental violence in childhood can affect social relationships in adolescence and adulthood by normalising violent behaviour. In particular, women who witnessed violence at home were related to emotional problems (anxiety, depression and low self-esteem) and socialisation problems [20]. Women who were victims of sexual violence at an early age are more likely to be involved in risky conditions such as early sexual intercourse, and possible transmission of sexually transmitted infections [35].

Likewise, this study identified components related to all conditions of violence. Women who made their own decisions are more likely to suffer from VAW [36]. Added to this, the presence of children, limited educational access, poor work situations and self-esteem problems create a moral conflict over the perception of family and personal responsibilities [37]. Likewise, experiencing abuse during childhood can normalize episodes of violence with the current partner, reducing personal support components [10]. Communities with less social openness can increase the risk of being a victim of different episodes of violence against women [38]. Alcohol consumption by the partner is a risk behavior strongly related to different levels of VAW. This would increase justifying attitudes of abuse on the part of the perpetrator and acceptance of the victims, promoting norms of masculinity that facilitate different types of violence scenarios [39]. Our findings evidenced a high relationship of disrespect from the aggressors with components of violence. This exercise of control allows the perpetrators to maintain power in a relationship by subjecting the partner to VAW strategies (e.g. physical or sexual) [40].

Help-seeking intention hardly manifest in episodes of psychological violence. Even living in community isolation and humiliation by the perpetrator leaves VAW victims with a feeling of emotional control and hopelessness [41–43]. This implies that help-seeking is a complex process in the natural cycle of violence [33, 44]. Although the body of evidence refers to the intention to help-seeking to sociodemographic variables and norms in a couple, these factors can be considered a set of dispositional variables [33, 45]. The systematic review by Barrett, Peirone, and Ho [35] has demonstrated cultural differences in help-seeking, identifying that Caucasian women might be more willing to help-seeking for VAW programs and services than other cultural groups (African and Latin women). However, our findings show that one of the main conditions for help-seeking is the presence of both types of violence (physical and sexual). This is due to the greater recurrence of help-seeking in health and judicial institutions due to the seriousness of their injuries [35].

Episodes of violence against women can increase due to patriarchal beliefs, poverty, lack of education and high birth rates, which are characteristics of some Peruvian regions. The high presence of VAW was found in the regions of the Peruvian highlands of Puno, Apurímac, Cusco and coast region of Piura. However, the Sierra region has between 50% and 70% Quechua-speaking inhabitants [46]. These contexts also share patriarchal beliefs that can devalue women, emerging structural inequalities manifesting in conditions of poverty and discriminating side effects on mental health, increasing women’s vulnerability to violence [8, 47]. These regions present wide wage gaps due to lack of access to education, with a more remarkable recurrence in the case of women [48]. This increases access to low-paid jobs that hinder economic independence towards exclusive dedication to children [47, 48]. These conditions facilitate a high birth rate, with more than ten children per Family. Added to the above, there is a high consumption of alcohol by the aggressors that allows the maintenance of episodes of violence and inequality of opportunities [8, 47, 48]. These findings underscore the need for interventions that address the root causes of VAW, including structural inequalities and discriminatory beliefs and practices.

### Public health relevance

Exposure to high VAW influences different areas of health, economic and social with greater variation in rural sectors. Countries with a high prevalence of VAW, such as Peru, could take into account the integration of effective models in mental health of community intervention in order to emphasize awareness-raising activities for the identification and reduction of conditions of violence in vulnerable stages (childhood and adolescence) [16, 17]. Despite the fact that health programs place greater emphasis on prevention, these results could help us identify paths based on gender inequality, structural factors, and inequitable regulations in order to reduce failures in communication and care for women victims of violence or other experiences of violence associated with gender [8, 35, 41]. It is important to take into account that the main sources of violence are found in the Peruvian highlands where the normalization of violence, language barriers, and lack of literacy in health services is high [46–48]. With a correct base of information and taking into account these structural factors, these conditions of violence against women could be addressed effectively.

### Strengths and limitations

Our dataset (ENDES) is based on the Demographic and Health Surveys (DHS) model [25] which has a valid and widely supported methodology that makes it possible a strong external validity (national representativeness). We confirmed the validity and reliability of the violence measurement. Our findings have significant implications for policy and practice and should be considered in the development of evidence-based interventions to prevent and address VAW. Among limitations, other variables that could help to better understand VAW were unavailable and could not be included in the analysis, such as information on the perpetration of violence in the home of origin, duration of exposure to violence, consumption of psychoactive substances, and other demographic variables (e.g., current type of employability).

## Conclusion

Women who reported having witnessing inter-parental violence in childhood are more likely to experience Violence Against Women (VAW) by their current partner. Likewise, physical and sexual violence would have a greater impact on these witnessing inter-parental violence in childhood, and when physical and sexual violence jointly occurred women were more help-seeking. The southern region of Peru is identified as an area of high vulnerability for women. It is crucial to promote educative and community-based programs aimed at the prevention and early recognition of VAW.

### Electronic supplementary material

Below is the link to the electronic supplementary material.

Supplementary Material 1

## Data Availability

The datasets generated and/or analyzed during the current study are available in https://zenodo.org/record/8253233. This database can also be obtained directly from ENDES with the following link https://proyectos.inei.gob.pe/microdatos/.
