# Supplementary material for: Witnessing inter-parental violence in childhood and help-seeking behaviours in violence against women in Peru
Source: BMC Public Health. 2024 Apr 12;24:1022. doi: 10.1186/s12889-024-18467-0 (PMC11015581; doi:10.1186/s12889-024-18467-0)
Supplement: Supplementary file 1 — Supplementary Material 1 [file 12889_2024_18467_MOESM1_ESM.docx]

# **Supplementary material**

### **Models of physical/sexual violence and global violence**

Table S1. Association between witnessing inter-parental violence in childhood and Physical/sexual and global violence, ENDES 2019 (N = 14,256)

|  | Physical and Sexual | | | | Global Violence | | | |
| --- | --- | --- | --- | --- | --- | --- | --- | --- |
|  | PR (CI 95%) | *p* | aPR (CI 95%) | *p* | PR (CI 95%) | *p* | aPR (CI 95%) | *p* |
| **witnessing inter-parental violence in childhood** | |  |  |  |  |  |  |  |
| Not | ref. |  | ref. |  | ref. |  | ref. |  |
| Yes | 1.40(1.09 - 1.80) | 0.009 | 1.33(1.07 - 1.65) | 0.009 | 1.31(1.23 - 1.39) | <0.001 | 1.23(1.16 - 1.30) | <.001 |
| **Age** |  |  |  |  |  |  |  |  |
| 15-28 | ref. |  | ref. |  | ref. |  | ref. |  |
| 29-35 | 1.31(0.94 - 1.83) | 0.112 | 1.36(0.99 - 1.87) | 0.058 | 1(0.94 - 1.07) | 0.941 | 1.01(0.94 - 1.08) | 0.857 |
| 36-49 | 1.59(1.18 - 2.13) | 0.002 | 1.55(1.12 - 2.15) | 0.008 | 1.08(1.01 - 1.15) | 0.029 | 1.07(0.99 - 1.16) | 0.067 |
| **Level Education** | |  |  |  |  |  |  |  |
| No Education/Primary | ref. |  | ref. |  | ref. |  | ref. |  |
| Secondary | 0.78(0.6 - 1.02) | 0.068 | 1.08(0.85 - 1.39) | 0.527 | 1.05(0.98 - 1.12) | 0.165 | 1.08(1 - 1.160) | 0.048 |
| Higher Education | 0.53(0.37 - 0.77) | 0.001 | 1.18(0.77 - 1.82) | 0.441 | 0.82(0.76 - 0.89) | <.001 | 0.96(0.87 - 1.05) | 0.377 |
| **Marital Status** | |  |  |  |  |  |  |  |
| Married | ref. |  |  |  | ref. |  | ref. |  |
| Cohabiting | 1.04(0.8 - 1.36) | 0.764 |  |  | 1.1(1.03 - 1.18) | 0.004 | 1.07(1.01 - 1.14) | 0.023 |
| **Economic level** | |  |  |  |  |  |  |  |
| Very Poor/Poor | ref. |  | ref. |  | ref. |  | ref. |  |
| Medium | 0.78(0.56 - 1.1) | 0.16 | 0.86(0.63 - 1.18) | 0.36 | 1.01(0.94 - 1.08) | 0.809 | 1.04(0.97 - 1.12) | 0.25 |
| Rich/Very rich | 0.61(0.4 - 0.92) | 0.018 | 0.68(0.43 - 1.08) | 0.105 | 0.90(0.84 - 0.97) | 0.004 | 1.03(0.95 - 1.11) | 0.493 |
| **Currently working** | |  |  |  |  |  |  |  |
| Not | ref. |  |  |  | ref. |  | ref. |  |
| Yes | 1.20(0.89 - 1.62) | 0.221 |  |  | 1.09(1.03 - 1.16) | 0.003 | 1.08(1.02 - 1.15) | 0.008 |
| **Area of residence** | |  |  |  |  |  |  |  |
| Coast | ref. |  | ref. |  | ref. |  | ref. |  |
| Highlands | 1.32(0.99 - 1.77) | 0.058 | 1.06(0.79 - 1.41) | 0.694 | 1.16(1.1 - 1.23) | <0.001 | 1.10(1.04 - 1.17) | 0.001 |
| Jungle | 1.28(0.95 - 1.73) | 0.106 | 0.98(0.74 - 1.29) | 0.879 | 0.99(0.93 - 1.06) | 0.785 | 0.92(0.86 - 0.99) | 0.019 |
| **Place of origin** | |  |  |  |  |  |  |  |
| Urban | ref. |  | ref. |  | ref. |  |  |  |
| Rural | 1.48(1.19 - 1.84) | <.001 | 1.11(0.85 - 1.44) | 0.45 | 1.01(0.96 - 1.06) | 0.800 |  |  |
| **Health Insurance** | |  |  |  |  |  |  |  |
| Not | ref. |  |  |  | ref. |  |  |  |
| Yes | 1.07(0.74 - 1.54) | 0.736 |  |  | 1.05(0.97 - 1.14) | 0.215 |  |  |
| **Differences in educational level** | | |  |  |  |  |  |  |
| Both with the same level | ref. |  |  |  | ref. |  |  |  |
| Woman with a higher level | 1.07(0.72 - 1.59) | 0.738 |  |  | 0.98(0.9 - 1.07) | 0.698 |  |  |
| Men with a higher level | 1.12(0.85 - 1.47) | 0.434 |  |  | 1.04(0.97 - 1.11) | 0.318 |  |  |
| **Number of children** | |  |  |  |  |  |  |  |
| None | ref. |  | ref. |  | ref. |  | ref. |  |
| 1-2 Children | 1.74(0.62 - 4.86) | 0.293 | 1.75(0.69 - 4.42) | 0.24 | 1.21(1.01 - 1.45) | 0.034 | 1.11(0.95 - 1.3) | 0.200 |
| 3-4 or more children | 2.75(0.98 - 7.66) | 0.054 | 1.96(0.75 - 5.13) | 0.168 | 1.44(1.2 - 1.72) | <0.001 | 1.21(1.02 - 1.43) | 0.029 |
| 5 or more | 4.08(1.45 - 11.46) | 0.008 | 2.42(0.89 - 6.58) | 0.083 | 1.39(1.16 - 1.67) | <0.001 | 1.12(0.94 - 1.34) | 0.217 |
| **Age of start of cohabitation** | | |  |  |  |  |  |  |
| 19-21 años | ref. |  | ref. |  | ref. |  | ref. |  |
| ≤ 18 years old | 1.39(1.07 - 1.82) | 0.014 | 1.21(0.96 - 1.52) | 0.107 | 1.06(1 - 1.13) | 0.059 | 1.05(0.98 - 1.11) | 0.161 |
| 22 and over | 0.79(0.53 - 1.17) | 0.239 | 0.77(0.52 - 1.13) | 0.179 | 0.83(0.76 - 0.9) | <0.001 | 0.88(0.81 - 0.96) | 0.003 |
| **Partner drinks alcohol** | |  |  |  |  |  |  |  |
| Does not drink | ref. |  | ref. |  | ref. |  | ref. |  |
| Drinks but does not get drunk | 0.51(0.28 - 0.96) | 0.036 | 0.61(0.33 - 1.13) | 0.117 | 0.97(0.86 - 1.08) | 0.555 | 0.99(0.89 - 1.1) | 0.870 |
| Drinks and gets drunk sometimes | 1.23(0.82 - 1.85) | 0.32 | 1.20(0.81 - 1.78) | 0.354 | 1.23(1.13 - 1.34) | <0.001 | 1.19(1.09 - 1.29) | <.001 |
| Drinks and gets drunk often | 2.34(1.46 - 3.75) | <.001 | 1.71(1.09 - 2.69) | 0.02 | 2.1(1.93 - 2.28) | <0.001 | 1.69(1.54 - 1.85) | <.001 |
| **Decision making** | |  |  |  |  |  |  |  |
| Joint decision | ref. |  | ref. |  | ref. |  | ref. |  |
| Woman's predominant decision | 1.56(1.06 - 2.28) | 0.024 | 1.26(0.89 - 1.78) | 0.2 | 1.26(1.18 - 1.35) | <0.001 | 1.18(1.11 - 1.26) | <.001 |
| Man's predominant decision | 1.54(0.92 - 2.58) | 0.104 | 1.25(0.78 - 2.01) | 0.354 | 1.21(1.08 - 1.36) | 0.001 | 1.17(1.05 - 1.32) | 0.006 |
| Split decision | 1.62(0.98 - 2.68) | 0.061 | 1.39(0.87 - 2.2) | 0.168 | 1.34(1.22 - 1.47) | <0.001 | 1.29(1.18 - 1.41) | <.001 |
| **Couple communication** | |  |  |  |  |  |  |  |
| Yes | ref. |  | ref. |  | ref. |  | ref. |  |
| Not | 3.28(2.49 - 4.31) | <.001 | 1.15(0.84 - 1.59) | 0.386 | 1.61(1.51 - 1.73) | <0.001 | 1.13(1.01 - 1.25) | 0.031 |
| **Respect by the partner** | |  |  |  |  |  |  |  |
| Yes | ref. |  | ref. |  | ref. |  | ref. |  |
| Not | 3.02(2.68 – 3.41) | <.001 | 1.96(1.69 – 2.27) | <0.001 | 1.76(1.66 - 1.88) | <0.001 | 1.35(1.21 - 1.5) | <.001 |
| **Parental agression** | |  |  |  |  |  |  |  |
| Not | ref. |  | ref. |  | ref. |  | ref. |  |
| Yes | 1.48(1.24 - 1.78) | <.001 | 1.37(1.15 - 1.62) | <.001 | 1.31(1.23 - 1.39) | <.001 | 1.34(1.22 - 1.47) | <.001 |

***Note***: n = 14,256. p < .001, PR: crude prevalence ratio, aPR: adjusted prevalence ratio. The final model was adjusted by variables that were associated

with the crude model for the three types of violence: age, educational level, marital status, socioeconomic level, current working, area of residence,

place of origin, health insurance, the difference in educational level, number of children, age of beginning of cohabitation, partner drinks alcohol,

decision making in the couple, couple communication, parental aggression and respect by the partner.

### **Validity of components VAW**

The report of the indicators of VAW was presented with the acceptance of aggression in at least one question; Likewise, the alpha and omega were evaluated for the reliability analysis, reporting acceptable values (0.791 and 0.761, respectively) and for the validity analysis, the confirmatory factor analysis (CFA) was used, presenting goodness-of-fit indices such as the comparative fit (CFI), Tucker-Lewis incremental fit (TLI), mean squared error of approximation (RMSEA); given that CFI and TLI to be greater than 0.90. RMSEA were less than 0.08.

Table S2. Validity of violence against woman model

|  | CFI | TLI | RMSEA | CI 90% |
| --- | --- | --- | --- | --- |
| model^a^ | 0.983 | 0.980 | 0.034 | (0.033 - 0.036) |
| model^b^ | 0.960 | 0.958 | 0.026 | (0.025 - 0.027) |

*Note*: model^a^ = ordered-categorical CFA model; model^b^ = complex survey CFA

### **Levels of VAW**

Percentiles were used for the cut-off points of the different types of violence.

Table S3. Cut-off points of the levels according to types of violence in the maps generated from the percentile distributions of the ENDES 2019

| Level | PSV | PV | SV | GV |
| --- | --- | --- | --- | --- |
| Low | 34.36- 40.50 | 15.46- 18.01 | 3.15- 3.70 | 39.85- 47.15 |
| Middle | 40.51- 48.80 | 18.02- 25.47 | 3.71- 5.51 | 47.16- 51.49 |
| High | 48.81- 58.35 | 25.48- 31.56 | 5.52- 7.31 | 51.50- 59.84 |
| Very high | 58.36-64.30 | 31.57- 38.22 | 7.32-11.28 | 59.85- 69.04 |

*Note*. PSV = psychological violence; PV = physical violence; SV = sexual violence; GV = Global violence. Percentiles were used for the cut-off points of the different types of violence.
